# Association between daily gait speed patterns and cognitive impairment in community-dwelling older adults

**DOI:** 10.1038/s41598-023-29805-4

**Published:** 2023-02-16

**Authors:** Kanako Seo, Naoto Takayanagi, Motoki Sudo, Yukari Yamashiro, Ippei Chiba, Keitaro Makino, Sangyoon Lee, Yoshifumi Niki, Hiroyuki Shimada

**Affiliations:** 1 Tokyo Research Laboratories, Kao Corporation, 2-1-3 Bunka, Sumida-Ku, Tokyo, 131-8501 Japan; 2 Department of Preventive Gerontology, Center for Gerontology and Social Science, National Center for Geriatrics and Gerontology, 7-430 Morioka, Obu, Aichi 474-8511 Japan; 3 Tohoku Medical Megabank Organization, Tohoku University, 2-1 Seiryo-Machi, Aoba-ku, Sendai, Miyagi 980-8573 Japan

**Keywords:** Geriatrics, Public health

## Abstract

Gait speed over a short distance is associated with cognitive impairment in older adults. Recently, daily gait speed has been assessed using accelerometers. However, because daily gait speed is only weakly correlation with gait speed over a short distance, its association with cognitive impairment needs to be investigated. The present study compared the daily gait speed patterns of normal cognition (NC), mild cognitive impairment (MCI), and general cognitive impairment (GCI) subjects measured every 3 h for two weeks using accelerometers. A total of 1959 participants were classified into the NC (N = 1519), MCI (N = 353), and GCI groups (N = 87). The results showed that the average daily gait speed of the GCI group was significantly lower than that of the NC group (*p* = 0.03). Furthermore, the average daily gait speeds of the MCI and NC groups were the same. However, the average daily gait speed of the MCI group during a specific time (12–15 o'clock) was significantly lower than that of the NC group (*p* < 0.01). These results suggest that changes in daily patterns may be detected by measuring daily gait speed, which depends on the degree of cognitive function.

## Introduction

Dementia is a syndrome that results in cognitive decline more than that expected with biological aging. According to the World Health Organization, dementia primarily affects memory, thinking, orientation, comprehension, calculation, learning capacity, language, and judgement^1^. Once dementia develops, it is difficult to cure or reduce it^2^; thus, detecting this syndrome as early as possible and decelerating its progression are important.

Gait dysfunction is considered one of the indicators of dementia^2^. The present study focused on gait speed as a parameter for monitoring gait function. Gait speed is generally considered to be an important marker of physical function decline in older adults^3^. Buracchio et al. reported that gait speed was associated with cognitive impairment^4^. In this study, out of 204 healthy older adults with an average of nine years of follow-up, 46% converted to mild cognitive impairment (MCI), and a change point was identified where acceleration in the decline of gait speed occurred approximately 12.1 years before the onset of MCI (95% CI: 8.1, unknown for left censoring), adjusted for age, education, sex, APOE-ε4 genotype, baseline speed, stroke, and depression.

Furthermore, Dumurgier et al. conducted a nine-year follow-up study of 3,663 older adults for incident dementia^5^. They reported that lower gait speed (< 100 cm/s) was associated with an increased risk of dementia (hazard ratio = 1.70, 95% CI 1.28, 2.26), adjusted for age, sex, marital status, education, number of unhealthy behaviors, height, BMI, Trail Making Test (TMT)-B score, depressive symptoms, psychotropic drugs, trauma, chronic conditions, cardiovascular disease, and the ApoE ε4 allele. These results support the finding that there is an association between gait speed and cognitive function, even after accounting for the effects of covariates. Therefore, by regularly monitoring the decrease in gait speed, older adults at high risk of cognitive impairment can be screened at an early stage.

Although gait speed is an important indicator of functional ability, various measurement protocols are used in clinical settings^6^. A systematic review of the relationship between gait speed and cognitive function reported that the most commonly adopted distance for measuring gait speed is a 6-m path including acceleration and deceleration phases^7^. In this experimental condition, because a subject can also temporarily increase the speed, the original gait speed may not be accurately evaluated, depending on the subject. To solve this problem, Dodge et al. continuously measured gait speed indoors (in-home gait speed) using passive infrared sensors to investigate its relationship with mild cognitive impairment (MCI)^8^. However, because this method is limited to evaluating gait speed indoors, outdoor activities cannot be considered. Another systematic review reported that physical activity during leisure time is associated with a reduced risk of dementia^9^. Because physical activities are generally considered to be outdoor activities, the relationship between gait speed and cognitive impairment becomes clearer by measuring gait speed including outdoor activities of elderly individuals.

Recently, it has become possible to measure gait speed in free-living conditions (daily gait speed) using a triaxial accelerometer^10–12^. Daily gait speed is known to decrease with age and gait speed at a short distance^11^, and it is associated with 2-year incident disability^13^. However, to our best knowledge, the relationship between gait speed patterns and cognitive function has been examined only in the above-mentioned study on in-home gait speed^8^. Using an accelerometer, daily gait speed including outdoor activities can be measured regardless of the measurement location. A previous study reported that daily physical patterns are associated with the early stage of Alzheimer's disease in older adults^14^. Therefore, we hypothesized that daily gait speed patterns are also associated with cognitive impairment in older adults. The present study classified community-dwelling older adults into general cognitive impairment (GCI), MCI, and normal cognition (NC) groups. Moreover, it compared the daily gait speed patterns of these groups measured for two weeks and examined the potential of monitoring daily cognitive decline using an accelerometer.

## Results

Table 1 reports the baseline characteristics, total number of steps per day, and average daily gait speed results of the three groups (NC, MCI, and GCI groups). One-way analysis of variance (ANOVA) showed the significant differences between the three groups in terms of the age (*p* < 0.01, *η*^2^ = 0.026) and self-reported education history (*p* < 0.01, *η*^2^ = 0.018). Furthermore, a significant difference was observed in terms of the average daily gait speed (*p* = 0.03, *η*^2^ = 0.013), and a post hoc comparison showed that the GCI group was significantly slower than the NC group (*p* = 0.03). Conversely, no significant difference was observed between the three groups (*p* = 0.12, *η*^2^ = 0.002) in terms of the total number of steps.

**Table 1:** Demographics of NC, MCI, and GCI groups.

Characteristics	Total (n = 1959)	NC (n = 1519)	MCI (n = 353)	GCI (n = 87)	P value	Effect size	Post hoc
Age, years	70.1 ± 6.1	69.8 ± 6.1	70.2 ± 6.0	74.6 ± 5.9	< 0.01*	*η*^*2*^ = 0.026	NC, MCI < GCI
Sex (ref: female)	1171 (59.8)	921 (60.6)	205 (58.1)	45 (51.7)	0.20	*V* = 0.041	n.s
BMI, kg/m^2^	23.4 ± 3.2	23.4 ± 3.1	23.3 ± 3.4	23.5 ± 3.4	0.84	*η*^*2*^ < 0.001	n.s
MMSE, score	27.8 ± 2.2	28.2 ± 1.8	27.4 ± 1.9	22.4 ± 0.8	< 0.01*	*η*^*2*^ = 0.303	NC > MCI > GCI
Grip strength, kg	28.3 ± 7.6	28.5 ± 7.7	27.9 ± 7.4	27.6 ± 7.0	0.24	*η*^*2*^ = 0.002	n.s
Education, years	11.6 ± 2.4	11.7 ± 2.4	11.5 ± 2.3	10.1 ± 2.1	< 0.01*	*η*^*2*^ = 0.018	NC, MCI > GCI
GDS, score	2.6 ± 2.4	2.5 ± 2.4	2.7 ± 2.5	3.1 ± 2.5	0.04	*η*^*2*^ = 0.003	n.s
Prescribed medications, number	2.4 ± 2.3	2.4 ± 2.3	2.5 ± 2.3	3.1 ± 3.0	0.01	*η*^*2*^ = 0.004	n.s
Hypertension, number (%)	870 (44.4)	663 (43.6)	162 (45.9)	45 (51.7)	0.28	*V* = 0.036	n.s
Diabetes, number (%)	232 (11.8)	171 (11.3)	45 (12.7)	16 (18.4)	0.11	*V* = 0.047	n.s
Hyperlipidemia, number (%)	592 (30.2)	460 (30.3)	109 (30.9)	23 (26.4)	0.72	*V* = 0.018	n.s
Daily gait speed, cm/s	104.4 ± 9.6	104.6 ± 9.3	104.1 ± 10.2	101.9 ± 11.5	0.03*	*η*^*2*^ = 0.013	NC > GCI
Number of steps, steps/day	6767.6 ± 2861.5	6767.0 ± 2823.8	6910.4 ± 3067.5	6197.8 ± 2597.8	0.12	*η*^*2*^ = 0.002	n.s

Data are shown as mean ± standard deviation.

*BMI* body mass index, *GDS* geriatric depression scale, *MMSE* mini-mental state examination.

*p < 0.05, *η*^*2*^ > 0.01, *V* > 0.10.

**Figure 1:**
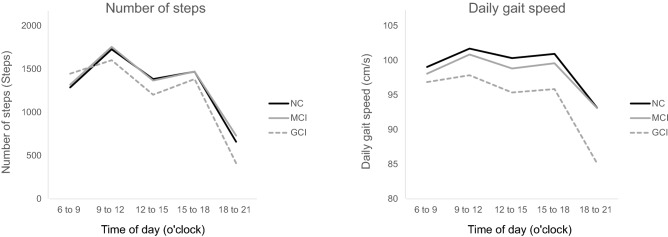
Average number of steps and average daily gait speed patterns measured every 3 h of normal cognition (NC), mild cognitive impairment (MCI), and general cognitive impairment (GCI) groups.

Table 2 summarizes and Fig. 1 shows the number of steps and average daily gait speed patterns measured every 3 h of the three groups. Two-way analysis of covariance (ANCOVA) (adjusting for the age, self-reported education history, GDS, and number of medications) showed significant main effects of the time of day (*p* < 0.01) and the groups (*p* < 0.01) on the number of steps. However, no interaction effect was observed between the time of day and the group (*p* = 0.31). The two-way ANCOVA also showed significant main effects of the time of day (*p* < 0.01) and the group (*p* < 0.01) on the average daily gait speed. Furthermore, a significant interaction effect was observed between the time of day and the group (*p* < 0.01). A post hoc comparison showed that the average daily gait speeds of the GCI group during 9–12 o'clock (*p* = 0.04) and 15–18 o'clock (*p* = 0.03) were significantly lower than those of the NC group. Moreover, the former during 18–21 o'clock (*p* < 0.01) were significantly lower than those of the NC and MCI groups. Furthermore, the average daily gait speeds of the MCI and GCI groups were significantly lower during 12–15 o'clock than that of the NC group (*p* = 0.05).

**Table 2:** Number of steps and daily gait speed every 3 h of NC, MCI, and GCI groups.

Characteristics	Time of day	Total (n = 1959)	NC (n = 1519)	MCI (n = 353)	GCI (n = 87)	Two-way ANCOVA	Post Hoc
Number of steps, steps	6–9 o'clock	1299.4 ± 1220.0	1286.5 ± 1217.5	1318.6 ± 1223.2	1445.1 ± 1253.7	Time of day < 0.01* Group < 0.01* Interaction = 0.09	n.s
	9–12 o'clock	1726.7 ± 931.6	1727.2 ± 917.6	1755.4 ± 999.2	1600.9 ± 889.1		n.s
	12–15 o'clock	1371.8 ± 771.1	1382.7 ± 769.5	1366.5 ± 796.8	1202.9 ± 675.0		n.s
	15–18 o'clock	1464.7 ± 905.6	1468.2 ± 883.1	1469.9 ± 965.3	1383.1 ± 1041.1		n.s
	18–21 o'clock	661.6 ± 672.0	659.8 ± 656.6	731.0 ± 763.5	412.6 ± 448.6		NC, MCI > GCI
Daily gait speed, cm/s	6–9 o'clock	98.8 ± 12.0	99.0 ± 11.8	98.1 ± 12.6	96.9 ± 13.7	Time of day < 0.01* Group < 0.01* Interaction < 0.01*	n.s
	9–12 o'clock	101.4 ± 9.6	101.7 ± 9.3	100.8 ± 9.7	97.8 ± 12.5		NC, MCI > GCI
	12–15 o'clock	99.8 ± 9.7	100.3 ± 9.3	98.8 ± 10.5	95.3 ± 12.0		NC > MCI > GCI
	15–18 o'clock	100.5 ± 10.6	100.9 ± 10.3	99.6 ± 11.0	95.8 ± 12.0		NC, MCI > GCI
	18–21 o'clock	92.8 ± 11.7	93.2 ± 11.2	93.1 ± 12.7	85.1 ± 13.0		NC, MCI > GCI

Data are shown as mean ± standard deviation.

*p-values reported from two-way repeated ANCOVA, adjusted for age, education, GDS (geriatric depression scale), and prescribed medications (*p* < 0.05).

## Discussion

The present study compared the daily gait speed patterns of the GCI, MCI, and normal cognition NC groups measured every 3 h. The results showed that the average daily gait speed of the GCI group was significantly lower than that of the NC group. Furthermore, there was no difference in the average daily gait speeds of the MCI and NC groups. However, the average daily gait speed of the MCI group during a specific time (12–15 o'clock) was significantly lower than that of the NC group.

Deterioration in gait function is associated with several adverse health outcomes, including falls^15,16^, disability^17^, and cognitive decline^5,18^. Researchers have investigated these relationships by measuring various gait parameters over short distances (short-term measurements). Zhou et al. predicted the fall status from 27 spatial–temporal gait characteristics measured at a 20 m distance using a wearable device with accelerometers, gyroscopes, and magnetometers in neurological patients^15^. Kikkert et al. quantified 23 gait outcomes measured at a 10 m distance and determined their discriminative power in healthy old adults and geriatric patients with and without cognitive impairment^19^. The outcomes of this study included gait speed, step regularity, cross-sample entropy, and frequency variability.

In free-living conditions (long-term measurements), Hausdorff et al. evaluated the quantity and quality of walking for seven days in older adults with MCI compared to age-matched controls^18^. In this study, participants wore a tri-axial accelerometer on the lower back for seven days, and their time spent walking (walking quantity), stride regularity and peak amplitude (walking quality) were measured. Since daily gait speed is one of the most versatile gait parameters, and various researchers have reported algorithms to measure this parameter using accelerometers^10–12^, we focused on daily gait speed among these gait parameters in this study. Furthermore, gait speed is also a familiar parameter when participants are given feedback on their measurements and are encouraged to improve. Differences in daily gait speed measured for 28 days at specific times among the three groups were observed, which suggests differences in lifestyle patterns between these groups. The present study focused on the daily gait speed (walking quality) and the number of steps (walking quantity). Combining daily gait speed with other gait outcomes, such as stride regularity and peak amplitude measured by Hausdorff et al.^18^ may clarify changes in activity patterns due to cognitive decline.

Although previous studies have reported that gait speed is associated with cognitive impairment^4,20^, it was unclear whether gait speed in daily living (i.e., daily gait speed) was associated with this impairment. A systematic review reported that gait speed has been assessed using paths of different lengths, ranging from 2.4 to 40 m^7^. Peters et al. compared the gait speeds over paths between 4 and 10 m for the same elderly participants. They observed that the gait speed over 4 m did not exhibit a sufficiently high degree of concurrent validity with that over 10 m^21^. Furthermore, in another study, when multiple trials of gait speed were measured, higher speeds were recorded in the later trials than those in the earlier trials, even over the same distance^22^. These previous studies suggest that gait speed results are probable to be affected by measurement conditions such as distance and/or number of trials. Conversely, daily gait speed can be continuously measured considering indoor and outdoor activities using an accelerometer, which may have low susceptibility to these effects. However, it remains unclear whether these parameters are associated with cognitive impairment. In the present study, the daily gait speed of the GCI group was observed to be significantly lower than that of the NC group, suggesting the possibility of screening GCI by monitoring daily gait speed.

Conversely, there was no difference in the average daily gait speeds of the MCI and NC groups. However, the average daily gait speed during a specific time (12–15 o'clock) of the MCI group was significantly than that of the MCI group. A previous study continuously measured gait speed indoors using an unobtrusive sensor system in a house to examine its relationship with MCI^8^. It compared the morning (6–15 o’clock) and evening (15–6 o’clock) gait speeds of MCI and healthy groups, and reported differences in these gait speeds. In the present study, differences were observed between the daily gait speed patterns of the MCI and NC groups measured by accelerometers (including both indoor and outdoor activities), consistent with the results of the above previous study. Therefore, the present results suggest that cognitive decline may cause changes in activity patterns, which may be detected by daily gait speed.

In particular, in this study, the daily gait speed of the MCI group during 12–15 o'clock was significantly lower than that of the NC group. Interestingly, no significant difference was observed between the number of steps of the groups during the same time. According to a survey on time use and leisure activities conducted by the Statistics Bureau of Japan^23^, time use is the time spent mainly preparing lunch, socializing with friends, and engaging in hobby activities for older adults. These can be considered as dual tasks, such as carrying luggage, thinking, and talking with others, which are different from a single task, such as only walking. The performance of a dual task requires more cognitive resources and higher activation in the prefrontal cortex than that of a single task^24,25^. Furthermore, for older adults with MCI, in a dual-task condition, gait speed is lower than in a single-task condition^26^. This may be the cause of the difference between the daily gait speeds of the MCI and NC groups measured at specific times.

The present study showed significant differences in average daily gait speed between the groups. Particularly, the average daily gait speed of the GCI group (101.9 ± 11.5 cm/s) was significantly lower than that of the NC group (104.6 ± 9.3 cm/s). In addition, the present study showed significant differences in the daily gait speed at different times of the day. For instance, participants for the MCI group walked fastest during 12–15 o'clock (100.8 ± 9.7 cm/s) and slowest during 18–21 o'clock (93.1 ± 12.7 cm/s). It should be noted that even among the groups in which significant differences were found, the differences in daily gait speed between (1) groups and between (2) time of the day was only approximately 5 cm/s. In addition, because an accelerometer was used to measure daily gait speed, measurement error is also expected to have an effect^27^. Conversely, the accelerometer enabled continuous monitoring of the participants’ daily gait speed^28^. In this study, participants who wore the accelerometer for more than 10 h during the 28-day measurement period were compared. Because daily gait speed is measured repeatedly in daily life, the standard error (SE) for each individual can be smaller, which is robust for measurement data. Therefore, the significant differences observed in this study are considered meaningful.

The present study observed differences in the daily gait speed at specific times between the MCI and NC groups. Daily gait speed can be monitored continuously daily using accelerometers, including differences in the time of day. Therefore, the present study suggests that by continuously monitoring the lifestyle patterns of older adults and detecting a decline in daily gait speed, it would be possible to screen and intervene at an early stage in older adults with a high risk of cognitive decline. A systematic review and meta-analysis^29^ that exercise programs with a short session duration and high frequency predicted greater cognitive effects in older adults with cognitive impairments. Based on this previous study, a combination of screening high-risk older adults through continuous monitoring of their daily gait speed and early intervention with short duration and/or high frequency for these older adults may help prevent further cognitive decline.

In terms of the number of steps per day, the present study observed no significant differences between the MCI and NC groups and between the GCI and NC groups. A previous study focusing on walking time reported that an MCI group has a shorter walking time than a control group^18^. Furthermore, Tayler et al. reported that the number of steps taken by older people with dementia was significantly lower than that by a control group^30^. One factor that may have contributed to the differences in the results of the present and previous studies is the difference in the physical function of the participants. In this study, the average number of steps per day of the GCI group was 6197.8 steps/day, which is much higher than that in a previous study (3307 steps/day)^30^. This may be attributed to the use of subjects who were not hospital patients, but instead older residents of the community, many of whom were physically healthy. Conversely, Aoyagi and Shephard reported that number of steps is associated with cognitive decline, based on a cohort study of healthy community-dwelling older adults^31^. Therefore, even in healthy older adults, continuous monitoring of the decline in the number of steps could be useful in predicting cognitive decline. Furthermore, in screening older adults for pre-frailty, it has been reported that combining the number of steps and daily gait speed improves screening accuracy^32^. Future studies are needed to determine whether the combination of daily gait speed and number of steps has a higher predictive performance of cognitive decline than the individual parameters.

This study had several limitations. First, the division of the participants into NC, MCI, and GCI groups was based on mini-mental state examination (MMSE) and National Center for Geriatrics and Gerontology–Functional Assessment Tool (NCGG-FAT) scores. Magnetic resonance imaging or other physiological measurements were not performed. Second, because the participants were older adults who voluntarily participated, they may have had a high level of both physical and mental health.

In conclusion, the present study compared the daily gait speed patterns of GCI, MCI, and NC subjects. The average daily gait speed of the GCI was significantly lower than that of the NC group. The average daily gait speed of the MCI group at a specific time (12–15 o'clock) was significantly lower than that of the NC group. These results suggest that changes in daily life patterns may be detected by measuring daily gait speed, which depends on the degree of cognitive function.

## Methods

### Participants

The present study was a part of the National Center for Geriatrics and Gerontology–Study of Geriatric Syndromes (NCGG-SGS), a cohort study aimed at establishing a screening system for geriatric syndromes^33^. In this study, 4167 older adults over 60 years of age residing in Takahama City, Aichi, Japan, participated. To minimize seasonal effects on activity levels, baseline assessments and daily data collection were conducted from September 2015 to June 2016. The study protocol was approved by the Research Ethics Committee of the National Center for Geriatrics and Gerontology (NCGG) (Approval Number 1440-3). Similar to previous studies^28,32^, all participants provided written informed consent by reading and signing a consent form approved by the institutional review board and agreed to wear accelerometers. After providing information about the study, the participants were given an option to decline participation, allowing them to withdraw directly or by proxy. This study was conducted in accordance with the Declaration of Helsinki guidelines.

### Baseline assessment

For each participant, a baseline assessment was conducted on only one day during the study period (September 2015–June 2016). Data on the age, sex, self-reported education history, and 15-item geriatric depression scale scores^34^ of the participants were collected. Data on stroke, depression, Parkinson’s disease, dementia, hypertension, diabetes, hyperlipidemia, and number of medications were recorded by face-to-face interviews with licensed nurses. In addition, the height and weight of each participant were measured, the body mass index was calculated, and the grip strength was measured using a Smedley-type handheld dynamometer (Grip-D, Takei Ltd., Niigata).

### Daily data collection

On the day of the baseline assessment, the participants were instructed to wear triaxial accelerometers (HW-100, Kao Corporation, Tokyo, Japan) on their waists at all times while awake, except when swimming or bathing, and to maintain their typical activities^28,32^. The participants were instructed to visit one of the 75 designated locations in Takahama City once every 30–40 days, where the accelerometer data were downloaded on a tablet PC via a near-field communication system (Sony Corporation, Tokyo, RC-380). The designated locations were chosen for their ease of access and included government-run facilities (e.g., community centers), gyms, drug stores, cafeterias, and beauty salons^28,32^.

### Daily gait speed and number of steps

The number of steps and daily gait speed of each participant were measured using the accelerometer. The daily gait speed was calculated from the physical intensity measured using the accelerometer. This device can measure 11 levels of physical intensity (0, 0.5, 1–9) every 4 s and estimate the gait speed based on these levels. The estimation accuracy of gait speed by this device is shown in Supplementary Fig. S1. In the present study, the data for 28 days from the day the participants started to wear the accelerometers were included in the analysis. Based on a systematic review^35^, a valid day was defined as any day on which the accelerometer was worn for ≥ 10 h, and participants with fewer than 15 valid days were excluded from the analysis. In addition to the total number of steps and average daily gait speed, the number of steps and average daily gait speed were measured every 3 h (i.e., 0–3, 3–6, 6–9, 9–12, 12–15, 15–18 o'clock, 18–21, and 21–24 o'clock). The number of steps and average daily gait speed of each participant during 6–9, 9–12, 12–15, 15–18, and 18–21 o'clock were analyzed to assess their activities in daily life.

### Cognitive evaluation

General cognitive functions, including orientation, registration, attention, calculation, recall, language, and copying, were evaluated using MMSE^36^. In addition, the NCGG–FAT^37^ was used to measure cognitive function in four domains: word memory (immediate recognition and delayed recall), attention (trail making test (TMT)-part A), executive function (TMT-part B), and processing speed (digit symbol substitution test). These four domains assessed cognitive impairments according to the standardized thresholds (within 1.5σ below the age- and education-specific means) established in the previous study^37^. The NCGG-FAT ranges from 0 to 4, with 0 meaning no impairment in any of the four domains and 1 to 4 meaning cognitive impairment.

In a previous study^38^, the participants were classified into three groups based on their MMSE and NCGG-FAT scores. First, the MMSE score was used to determine whether or not the participants were in the GCI group (MMSE score < 24 points). Second, the NCGG-FAT was used for the remaining participants to determine whether they were in the MCI or NC group. Participants with no impaired domain (i.e., none of the four domains was impaired) for the NC group and those with one, two, three, or four impaired domains for the MCI group were classified.

### Data analysis

Of the 4167 participants (1840 men and 2327 women), 363 participants with medical histories (240 strokes, 109 depression, 19 Parkinson's disease, and 8 dementia) and 42 participants who could not be determined owing to lack of MMSE and NCGG-FAT data were excluded from the analysis. Furthermore, 1803 participants who could not meet the criteria for accelerometer data were excluded from the analysis (i.e., they did not wear the accelerometer on their waist for ≥ 15 days for ≥ 10 h/day, during the 28 days after starting to wear the accelerometer). A total of 1959 participants were included in the final analysis: 1519 in the NC group, 353 in the MCI group, and 87 in the GCI group.

### Statistics

One-way ANOVA was conducted for continuous variables and chi-squared tests for categorical variables, to test the differences between the three groups (NC, MCI, and GCI groups) in terms of the baseline characteristics, total number of steps per day, and average daily gait speed. In addition, two-way ANCOVA was conducted to determine the significant differences between five time of days (6–9, 9–12, 12–15, 15–18, and 18–21 o'clock) and the three groups in terms of the number of steps and average daily gait speed, respectively. For these two-way ANCOVA tests, the variables used for adjustment were age, education level, GDS, and prescribed medications. Post hoc analyses of the ANOVA and ANCOVA results were performed using the Bonferroni test for the three groups. Bonferroni corrections were applied to minimize family-wise error, and adjusted *p*-values were used for multiple simultaneous comparisons. Differences in means were considered statistically significant if the *p* values were < 0.05, partial *η*^2^ values were > 0.01, and *V* values were > 0.10^39^. All statistical analyses were performed using the SPSS statistical software package (IBM SPSS Statistics Version 26, SPSS Inc., Chicago, IL, USA).

## Supplementary Information

Supplementary Information.

## Data Availability

The datasets generated and/or analyzed during the current study are not publicly available because of intellectual property reasons but are available on reasonable request. If you request data from this study, please contact the corresponding author via e-mail.
