# Supplementary material for: Association between daily gait speed patterns and cognitive impairment in community-dwelling older adults
Source: Sci Rep. 2023 Feb 16;13:2783. doi: 10.1038/s41598-023-29805-4 (PMC9935628; doi:10.1038/s41598-023-29805-4)
Supplement: Supplementary file 1 — Supplementary Information. [file 41598_2023_29805_MOESM1_ESM.docx]

**Supplementary information**

**Association between daily gait speed patterns and cognitive impairment in community-dwelling older adults**

**Kanako Seo^1*^, Naoto Takayanagi^1^, Motoki Sudo^1^, Yukari Yamashiro^1^, Ippei Chiba^2^, Keitaro Makino^2^, Sangyoon Lee^2^, Yoshifumi Niki^1^, Hiroyuki Shimada^2^**

^1Tokyo Research Laboratories, Kao Corporation, 2-1-3 Bunka, Sumida-ku, Tokyo 131-8501, Japan^

^2Department of Preventive Gerontology, Center for Gerontology and Social Science, National Center for Geriatrics and Gerontology, 7-430 Morioka, Obu, Aichi 474-8511, Japan^

^*seo.kanako@kao.com^

**Accuracy evaluation of gait speed measured by accelerometer**

This supplementary information presents the results of evaluating the accuracy of the triaxial accelerometer used to measure daily gait speed in the present study.

**Methods**

**Participants**

In the present study, 56 participants (28 men and 28 women) aged 25–59 years participated. Their mean age (± SD) was 40.2 ± 10.8 years, mean height was 166.0 ± 8.5 cm, mean body weight was 59.5 ± 9.90 kg, and mean body mass index was 21.5 ± 2.6 kg/m^2^. The study protocol was approved by the Human Research Ethics Committee of the Kao Corporation (No. S143-180129). All participants provided written informed consent by reading and signing a consent form approved by the institutional review board. This study was conducted in accordance with the Declaration of Helsinki guidelines.

**Gait speed measurement**

Gait speed measurements were conducted on a 26-m walkway including 3 m for acceleration and another 3 m for deceleration. Gait speed was measured at a 20-m position in the middle of the walkway. The actual gait speed was measured using a stopwatch, which is the gold standard. The gait speed was estimated by measuring using a triaxial accelerometer (HW-100, Kao Corporation, Tokyo, Japan). This accelerometer estimates daily gait speed based on physical intensity during walking.

The participants were instructed to wear the accelerometer on the right side of their waist. They were also instructed to walk on the 26-m walkway at three different paces:1) typical pace, 2) higher pace than typical, and 3) lower pace than typical. Gait speed was measured twice at each pace, and the data of six trials per participant (336 trials in total) were obtained.

**Statistics**

The relationship between the actual and estimated gait speeds was examined by calculating Pearson’s correlation coefficients (*r*). Differences in means were considered statistically significant if *p*-values were less than 0.05. Furthermore, the systematic error (SE) measured in the two different conditions was calculated as an index to evaluate the performance of the accelerometer.

*SE* = $\frac{1}{N} \sum_{i=1}^{N} (Actual gait speed-Estimated gait speed)$

All statistical analyses were performed using the SPSS statistical software package (IBM SPSS Statistics Version 26, SPSS Inc., Chicago, IL, USA).

**Results**

Supplementary Figure S1 shows the relationship between the actual and estimated gait speeds. A strong correlation was observed between these parameters (*r* = 0.906, *p* < 0.001). The SE between these parameters was 9.43 cm/s.


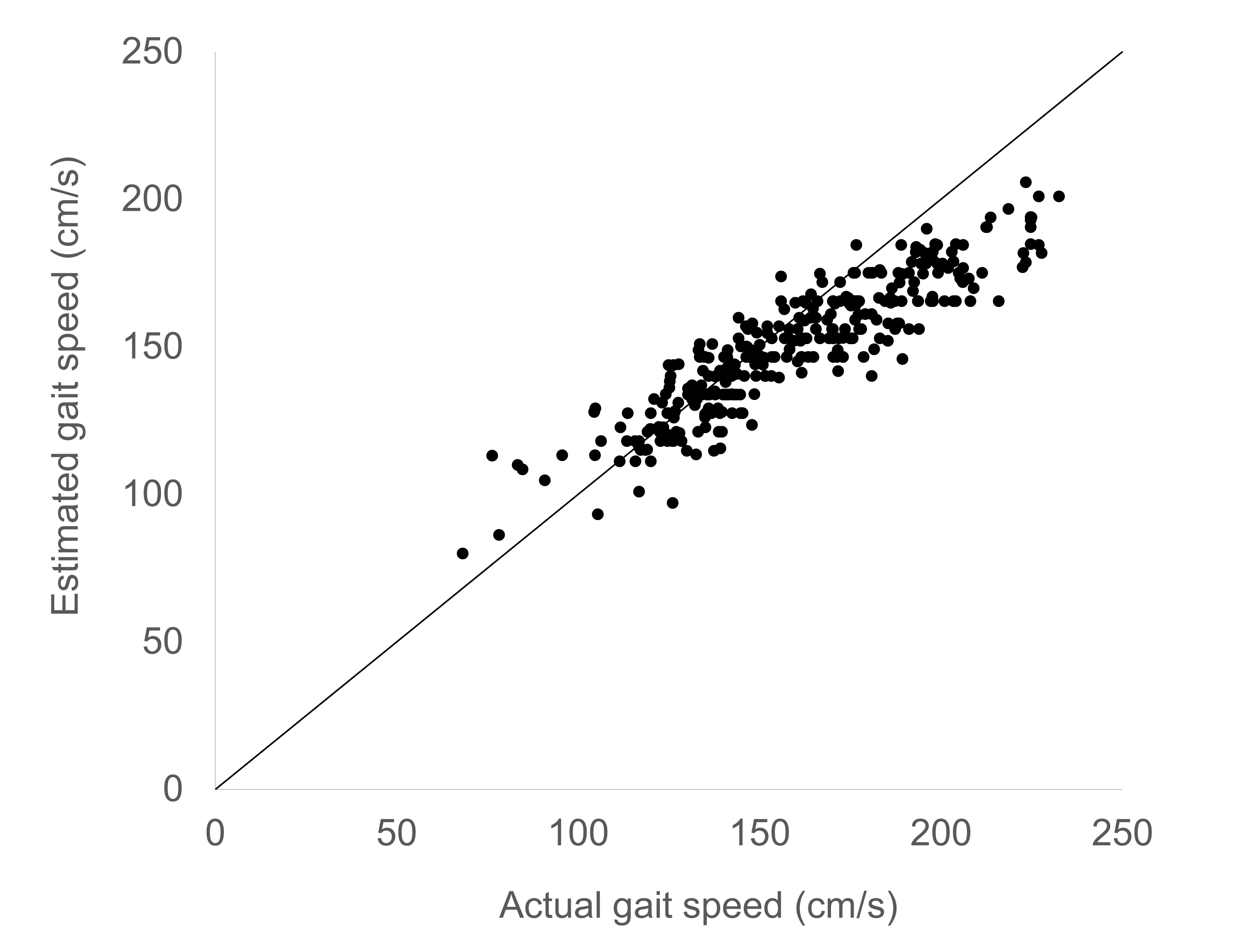


**Figure S1.** Accuracy evaluation of gait speed measured by triaxial accelerometer. Participants are instructed to walk along walkway at three types of paces: 1) typical pace, 2) higher pace than typical, and 3) lower pace than typical. Gait speed measurements at each pace are conducted twice. *r* = 0.906, *p* < 0.001; 46 participants × 6 trials = 336 trials.
